# Volcano plots in hydrogen electrocatalysis – uses and abuses

**DOI:** 10.3762/bjnano.5.96

**Published:** 2014-06-13

**Authors:** Paola Quaino, Fernanda Juarez, Elizabeth Santos, Wolfgang Schmickler

**Affiliations:** 1 PRELINE, Universidad Nacional del Litoral, Santa Fe, Argentina; 2 Institute of Theoretical Chemistry, Ulm University, D-89069 Ulm, Germany; 3 Faculdad de Matemática, Astronomía y Física, IFEG-CONICET, Universidad Nacional de Córdoba, Córdoba, Argentina

**Keywords:** electrocatalysis, hydrogen evolution, Sabatier’s principle, volcano curve

## Abstract

Sabatier’s principle suggests, that for hydrogen evolution a plot of the rate constant versus the hydrogen adsorption energy should result in a volcano, and several such plots have been presented in the literature. A thorough examination of the data shows, that there is no volcano once the oxide-covered metals are left out. We examine the factors that govern the reaction rate in the light of our own theory and conclude, that Sabatier’s principle is only one of several factors that determine the rate. With the exception of nickel and cobalt, the reaction rate does not decrease for highly exothermic hydrogen adsorption as predicted, because the reaction passes through more suitable intermediate states. The case of nickel is given special attention; since it is a 3d metal, its orbitals are compact and the overlap with hydrogen is too low to make it a good catalyst.

## Introduction

Sabatier’s principle [1] is one of the oldest rules in catalysis. For a two-step reaction passing through an adsorbed intermediate, like the hydrogen reaction, it states that the adsorption energy should be neither too high nor too low. If it is is too high (endothermic), adsorption is slow and limits the overall rate; if it is too low (exothermic), desorption is slow. In terms of hydrogen electrocatalysis it can be stated more precisely: at the equilibrium potential the free energy of adsorption of hydrogen from solution should be close to zero.

**Figure 1:**
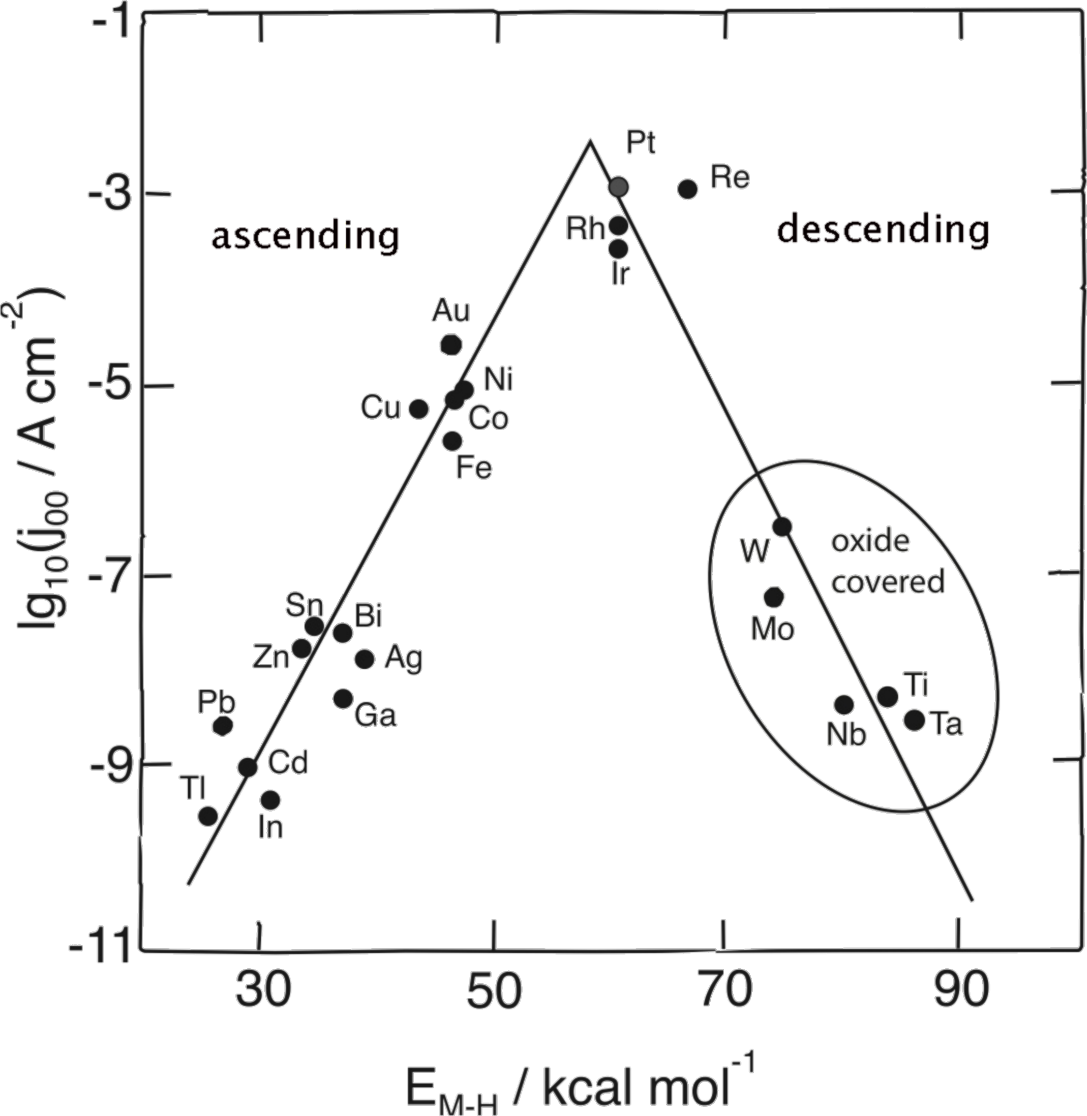
Trassati’s volcano plot for the hydrogen evolution reaction in acid solutions. *j*_00_ denotes the exchange current density, and *E*_MH_ the energy of hydride formation. Data taken from [5].

If Sabatier’s principle is the only factor that governs a reaction, a plot of the reaction rate versus the free energy of adsorption of the intermediate results in a volcano curve. Starting from a high, positive (endergonic) energy of adsorption Δ*G*_ad_, the rate at first rises with decreasing Δ*G*_ad_; this is the ascending branch of the volcano. Near Δ*G*_ad_ ≈ 0 the rate passes through a maximum, and then starts to decrease as Δ*G*_ad_ becomes more exergonic (descending branch). Still, experimental evidence for a volcano relation in heterogenous catalysis is scarce. In electrochemistry, Gerischer [2] and Parsons [3–4] were the first to point out that certain models for the hydrogen reaction predicted a volcano-like curve. However, it was Trasatti [5] who collected experimental data and constructed the first volcano curve for hydrogen evolution. Since experimental or theoretical data for hydrogen adsorption were not available at this time, he used the energy of hydride formation instead. His plot, which has been reproduced in many textbooks, is shown in Figure 1 and covers acid solutions. The reaction rate is expressed in terms of the exchange current density, which is proportional to the reaction rate at the equilibrium potential.

The ascending branch of Trasatti’s volcano plot is quite convincing; however, on the descending branch, there are only metals which are covered by an oxide film during hydrogen evolution, a fact that was not known at that time. Naturally, the presence of an oxide film reduces the reaction rate by several orders of magnitude. If we disregard the oxide-covered metals, there is no evidence for the descending branch.

In recent years, there has been much seismic activity, and several versions of volcano plots, not only for hydrogen evolution, have been constructed. In this article we shall critically consider the concepts behind and the experimental results for the hydrogen volcano plot, both in acid and in alkaline solutions. We will present our own ideas and show some new theoretical results for nickel, which in modern volcano plots is the only metal on the descending branch.

There is a parallelism between the concept of volcano plots in catalysis and outer sphere electron transfer reactions. According to Marcus’ theory [6] a plot of the reaction rate versus the reaction free energy Δ*G* should pass through a maximum when Δ*G* ≈ −*λ*, where *λ* is the energy of solvent reorganisation of the reaction, and fall off for more exergonic reactions; the descending branch is known as the Marcus inverted region. While there are many electron transfer reaction which clearly show the ascending branch, there are very few examples where the inverted region has been observed. We shall return to this point.

## Modern volcano plots

Before presenting a new version of the volcano plot, we would like to remind our readers of the mechanisms of hydrogen evolution and oxidation. In acid media, the first step in hydrogen evolution is always the Volmer reaction or electrochemical hydrogen adsorption:

[1]
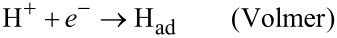


while for the second step there are two possibilities:

[2]
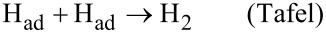


[3]
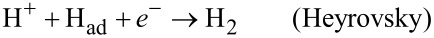


In alkaline solutions, the Volmer and Heyrovsky reactions are:

[4]
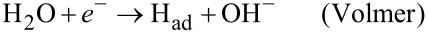


[5]
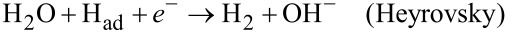


while the Tafel reaction stays the same.

Modern volcano plots, pioneered by the Nørskov group [7], use adsorption energies calculated by density functional theory (DFT). These are quite reliable for hydrogen adsorption – more so than experimental values – with an estimated error of ±0.1 eV. We have calculated these adsorption energies for a fair number of densely-packed metal surfaces, mostly fcc(111). In those cases, in which we considered the same metals, we obtained the same values as Nørskov et al. [7] within the usual DFT error. In contrast, the experimental values for the reaction rates measured by different groups sometimes vary by two orders of magnitude. The sources for our data are given in the appendix. We have not considered metals that are known be covered by oxide of hydroxide layers during hydrogen evolution.

**Figure 2:**
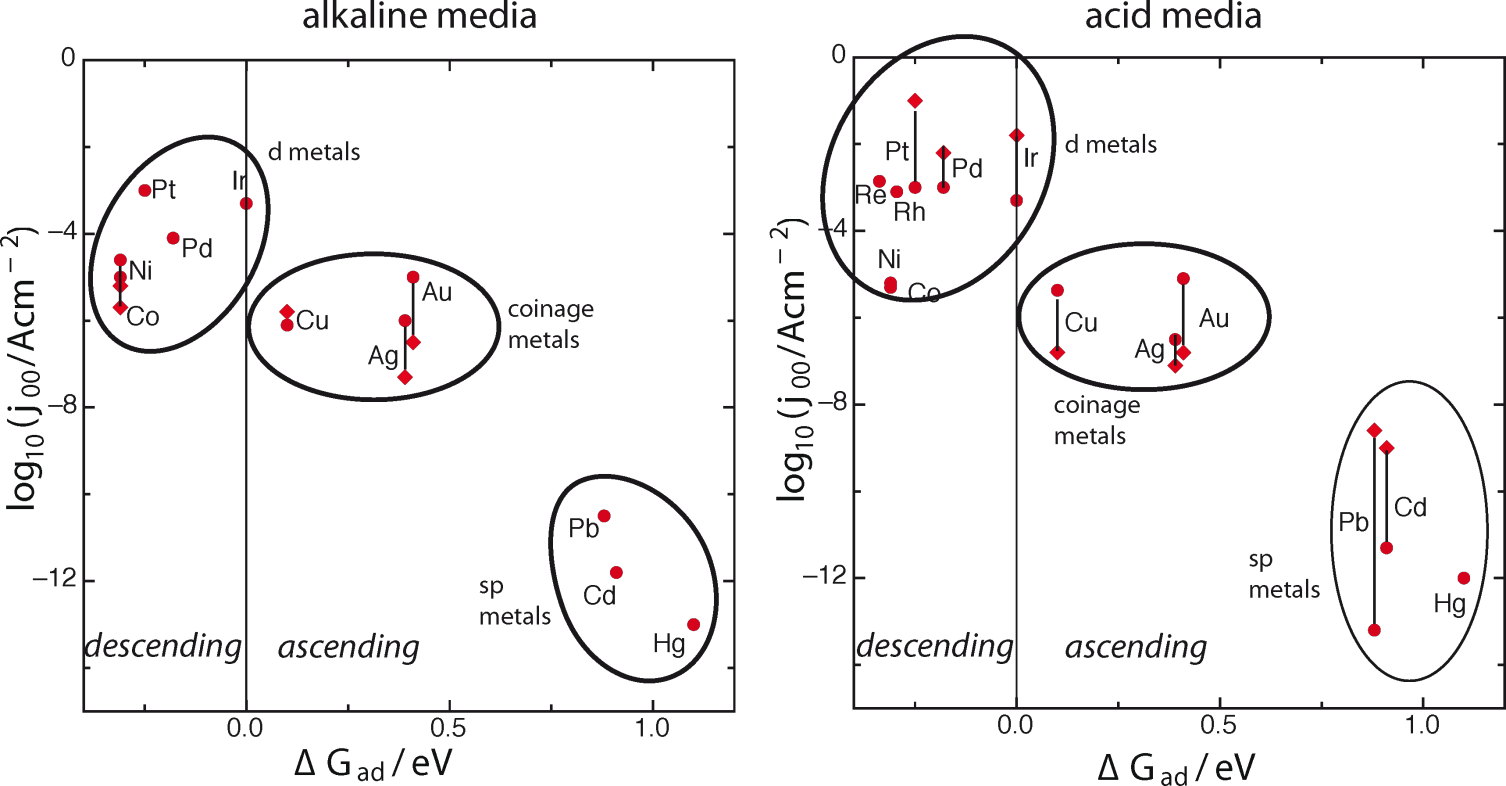
’Volcano’ plots for hydrogen evolution in acid and alkaline aqueous solutions. Note that ascending and descending branch are reversed with respect to Figure 1.

The resulting plots are shown in Figure 2 both for acid and for alkaline solutions. Wherever there is a significant spread of experimental data, we have indicated the corresponding error bars. There are more data for acid than for alkaline media, because the former are relevant for the most popular type of fuel cells, proton-exchange membrane (PEM) cells. Both plots look quite similar, but the fastest rates in acid solutions are somewhat faster than in alkaline. Neither of the plots bears any resemblance to a volcano, but there is a pronounced increase of the rate with decreasing (more favorable) Δ*G*_ad_ in the endergonic region. There is a clear separation into three groups: sp metals, which are the worst catalysts, coinage metals, which are intermediate, and the d metals, which contain the best catalysts, but also Ni and Co, which are mediocre.

A comparison with Trasatti’s plot for acid media shows an overall similarity, once the oxide covered surfaces have been discarded from the latter, but also a few significant deviations. Some of these changes are due to new experimental values, others are caused by the fact, that the trends in hydride formation energies used by Trasatti do not always follow the hydrogen adsorption energies. An example for the latter is the position of nickel, an example for the former is the higher rate at silver in our plot, which is probably due to better sample preparation.

## Discussion

Our group has developed its own theory of hydrogen electrocatalysis, based on a model Hamiltonian, quantum statistics and DFT, which we have reviewed in [8]. From our work we have derived three rules for a good catalyst:

- It should follow Sabatier’s principle, Δ*G* ≈ 0 at the equilibrium potential;
- have a d band which spans the Fermi level;
- have a strong and long-ranged interaction between the d band and the hydrogen 1s orbital. A long range is important, because the electron transfer to the proton occurs at a certain distance, of the order of 0.5 Å, from the adsorption site [9].

These three conditions are not independent, since the position of the d band and the interaction strength also affect the energy of adsorption. Nevertheless, they can sometimes act against each other, as we shall show below. We proceed to discuss the plots of Figure 2 in the light of these principles. It is convenient to consider the three groups separately.

### sp Metals

In the sp metals the d band lies so low that it plays no role in the bonding of hydrogen nor in electrocatalysis. This does not imply that the interaction of the d band with the adsorbed hydrogen is weak. For instance, in the case of Cd the interaction is sufficiently strong to produce nice bonding and antibonding peaks in the density of state (DOS) of the adsorbed hydrogen [10]. However, both bonding and antibonding states are filled, and hence this interaction does not contribute to the binding. In the absence of d band effects, we should expect this group of metals to follow Sabatier’s principle. For alkaline solutions this is clearly the case, while for acid solutions the situation is not quite so clear. The difficulty with this groups of metals is that measurable currents can only be obtained at high overpotentials, so that the determination of the exchange current density *j*_00_ requires an extrapolation over a large potential range. Further, on some metals like Pb and Cd there is a change in the slope of current–potential curves. Depending on which part of the curves are extrapolated, one obtains widely different values for *j*_00_. Trasatti’s [5] values are higher than those suggested by Petrii and Tsirlina [11] and nicely follow Sabatier’s principle, while according to the latter group the reaction is slower on Pb than on Hg.

Of all the metals that we have investigated by using DFT, Hg is unique in that hydrogen is adsorbed on top; on all other metals it adsorbs at hollow sites. Also, it has the highest (least favorable) energy of adsorption. It must also be stated that the experimental values are quite old. Nowadays, there is a frantic search for good catalysts, and nobody is interested in sp metals, even though the most common car battery, the lead battery, only works because lead is such a bad catalyst for hydrogen evolution. Also, mercury once plaid a pivotal role as the electrode material for polarography, which used to be an important analytical technique. In fact, the only Nobel prize that has so far been awarded to an electrochemist was to Heyrovsky because of his work on polarography. In any case, in Trasatti’s plot (Figure 1) the sp metals do follow Sabatier’s principle quite well, and our plot supports this in alkaline solutions, while the data in acid solutions at least do not contradict this. Also, the fact that on these metals the Volmer reaction is the rate determining step [12] is quite in line with Sabatier’s principle.

### Coinage metals

The three coinage metals are mediocre catalysts; the experimental values for the exchange current densities also have to be extrapolated, but not over such a large potential range as is the case for the sp metals. Older data suffer from inadequate preparations of the electrode surface; for Ag and Au we have only considered experiments where the electrode had been treated by flame annealing. The spread of experimental data is much less than for the sp metals, and within experimental error the rates are about the same on all three metals, both in acid and in alkaline solutions. On Cu and Au, the Volmer reaction determines the rate, while on Ag the Volmer and Heyrovsky step are quite similar [12–17]. The Tafel reaction plays no role.

**Figure 3:**
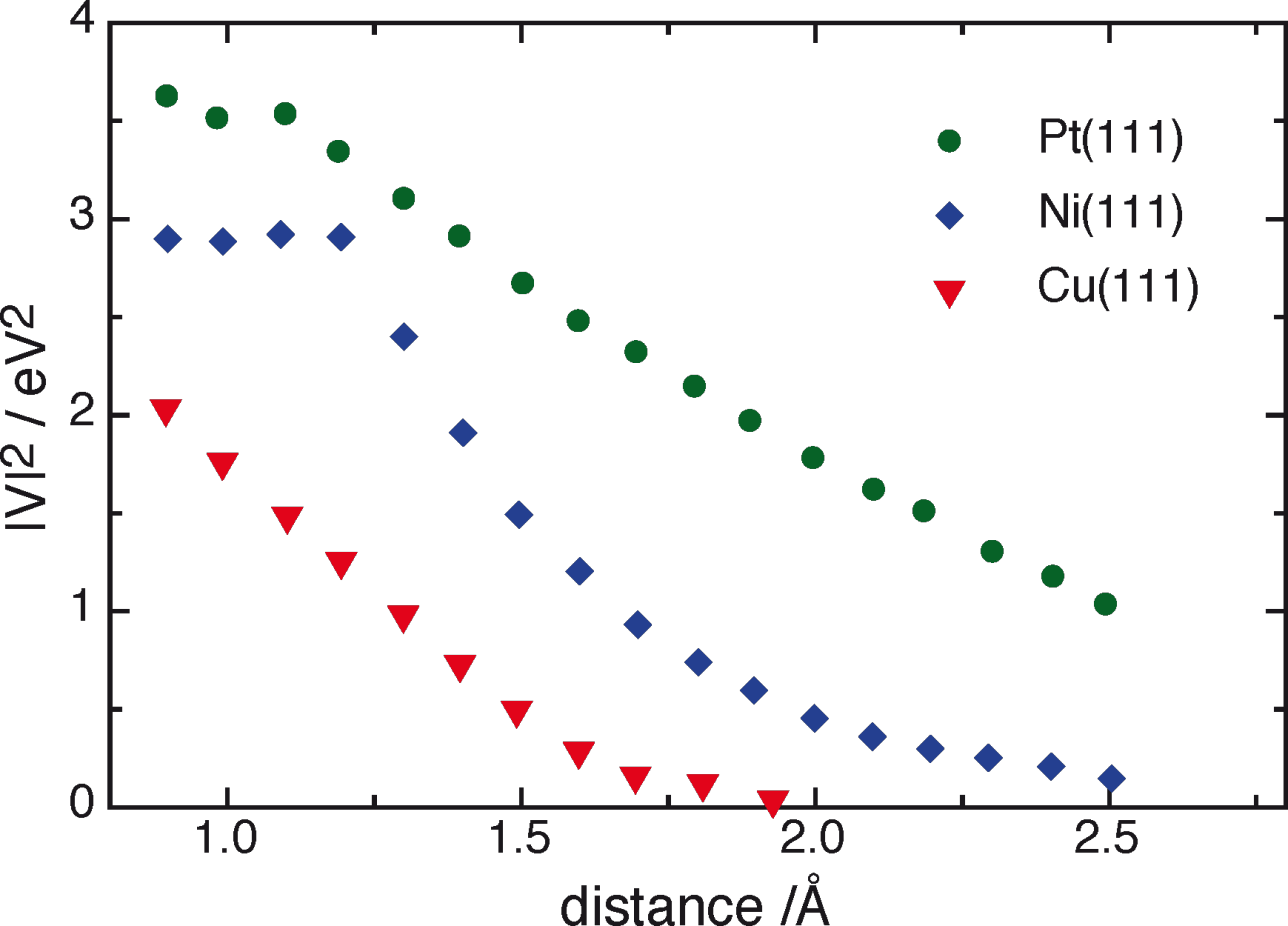
Square of the coupling constants between the H1s orbital and the d bands of Pt(111), Ni(111), Cu(111).

If the hydrogen evolution were governed by Sabatier’s principle alone, copper, with Δ*G*_ad_ = 0.1 eV, should be an excellent catalyst, better than platinum with Δ*G*_ad_ = −0.2 eV. Its d band does not span the Fermi level, but ends about 0.1 eV below. So its position is not optimal, but close enough to make a contribution to the binding of hydrogen. However, as a third row element the orbitals of copper are compact; therefore the overlap with the hydrogen 1s orbital is short-ranged. As shown in Figure 3, the interaction of the copper d band with hydrogen has almost dropped to zero at a distance of the order of 1.4 Å, where electron transfer typically occurs [10]. The fact that the rate of hydrogen evolution is roughly the same on the three coinage metals is due to two opposing effects: The position of the d band and the energy of adsorption become more favorable in the order Au < Ag < Cu, while the coupling becomes weaker. Thus, the overall rates of the coinage metals are not governed by Sabatier’s principle alone, and form a plateau rather than a volcano.

However, the difference in the rate constants for Cu(100) and Cu(111) in acid solutions, and also for Ag(100) and Ag(111), can be explained by Sabatier’s principle. In both cases, the rate is somewhat faster on the (111) than on the (100) surfaces [15,18–19], and the adsorption energy is also lower on the more compact surfaces [20]. Theses differences are so small that we could not show them in our plot, but they are well established.

### d Metals

By definition, the Fermi level of the d metals lies within the d band, so they fulfill at least one of our criteria for good catalysts. Indeed, with the exception of Co and Ni the rate is fast on all the metals that have been investigated – they are so fast that they are difficult to measure. In recent years, it has been claimed that the old values for Pt, Ir, Pd, as used by Trasatti [5] or Nørskov et al. [7], are too low because of mass transport limitations [21–22]. The new values correspond to the upper points in the error bars for these metals in Figure 2, while the lower points indicate the older values. None such measurements have been made for other metals of this group like Re and Rh, and the rates on these metals may well be higher than indicated. In any case, with the exception of Co and Ni, the rates are fast on these metals. They seem to be about one order of magnitude faster in acid than in alkaline solutions, and they do not follow a volcano shape. Thus their behavior is not governed by Sabatier’s principle alone.

We shall consider nickel and cobalt in detail later, and now focus on the other d metals. Starting from iridium, they ought to form the descending branch of the volcano, but obviously they do not. The reason is, that there are two distinct species: (1) The strongly adsorbed hydrogen, also called upd hydrogen (upd means *deposited at underpotentials*); it is the energy of this species that is generally used in volcano-type plots. (2) A weakly adsorbed species, also called opd hydrogen (opd means *deposited at overpotentials*). This topic is well reviewed in an article by Jerkiewicz [23]. With two states available, the reaction simply passes through the intermediate with the more favorable energy, and avoids the descending branch predicted by Sabatier’s principle. At polycrystalline metals there are even more sites and therefore more options.

The relation between the two species is not simple, since the energy of the weakly adsorbed hydrogen depends on the coverage with the upd species. The best investigated case is Pt(111) in acid solutions, where the strongly adsorbed hydrogen is clearly visible in the cyclic voltammogram at potentials above the onset of hydrogen evolution; the total coverage of this species reaches about 70% in this region. However, the weakly adsorbed species has also been detected by infrared spectroscopy above the hydrogen evolution region [24]. So the adsorption of the weakly adsorbed species sets in before the coverage with the other one is complete. This is important, because the two species repel each other, and with increasing coverage of upd hydrogen both the energy of the opd species and the activation energy for the Tafel reaction increase noticeably. Therefore, a DFT calculation for the free energy of adsorption of the weakly adsorbed species in the presence of a monolayer of upd hydrogen gives often quite high (unfavorable) values for the the adsorption energy of the former species. We have discussed this point in detail in a a recent communication [25], where we have also calculated the isotherms for both species of adsorbed hydrogen on Pt(111). In any case, the interaction between the two species makes it quite difficult to calculate the adsorption free energy of the true intermediate state by DFT.

In the introduction, we mentioned a similarity between the volcano plots predicted by Sabatier’s principle, and the free energy relation predicted by Marcus’ theory for outer sphere electron transfer. It was very difficult to prove the existence of the Marcus inverted region at high reaction free energies, because in most reactions the electron can be transferred to a state with a higher, and thus more favorable, energy. In fact, this is the reason why the Marcus inverted region cannot be observed at metal electrodes, where in the highly exothermic region the electron can pass to the multitude of empty states that lie above the Fermi level [26]. Similarly, it is difficult to find an electrode material that follows the descending branch of the volcano curves, because the metals with a strong affinity to hydrogen usually have more than one adsorbed states, and the reaction passes through the more favorable ones. Nickel and cobalt are the only metals that lie on the descending branch, and they are worth a special look.

### Nickel

**Figure 4:**
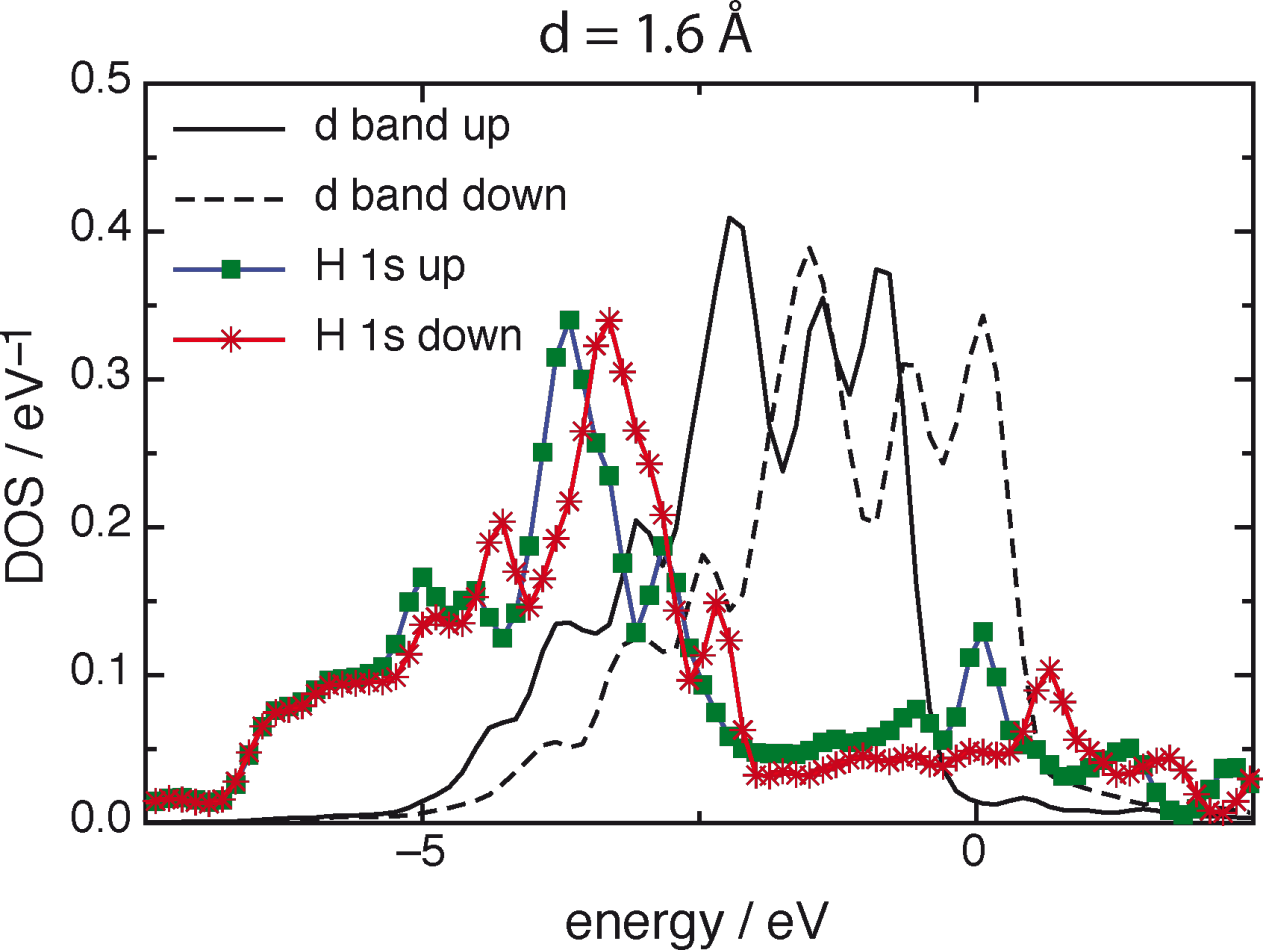
Densities of states of the d bands of Ni(111) and of the 1s spin orbitals of a hydrogen atom at a distance of 1.6 Å from the surface.

Nickel and cobalt are very similar, and we focus on Ni(111), which is the densest and most stable surface. Nickel is one of the few metals that are spin polarized, and the d bands for spin up and spin down are shifted with respect to each other, even though they have the same shape (see Figure 4). This has a marked effect on the spin polarization of a hydrogen atom in front of the surface. On the densest-packed surface of most metals, spin polarization of the H1s orbital vanishes at about 2.4 Å [10,27]. In contrast, on Ni(111) spin polarization persists to much shorter distances. As an example, we show the densities of states (DOS) at a distance of 1.6 Å. For the two spin states of H1s, the DOS have almost the same shape but are shifted with respect to each other. Each spin orbital interacts principally with its d band counterpart on nickel, and exhibits clear bonding and antibonding peaks. At shorter distances, the spin polarization of hydrogen vanishes gradually, and is absent when the atom is adsorbed at a distance of 0.9 Å.

Nickel is a 3d metal, in the same row as copper. This entails that its orbitals are compact, and the interaction with the hydrogen atom falls off rapidly with distance [25], even though the adsorption energy is quite favorable (see Figure 3). The most favorable adsorption site is the (111) hollow site. The next best site, which corresponds to weakly adsorbed hydrogen, is on top, has an adsorption energy of 0.9 eV, in the presence of a monolayer of strongly adsorbed hydrogen, and is thus highly unfavorable. Therefore hydrogen evolution must pass through the strongly adsorbed hydrogen, and thus follows Sabatier’s principle, while the other d metals (except cobalt) can escape by passing through intermediates with a higher energy.

**Figure 5:**
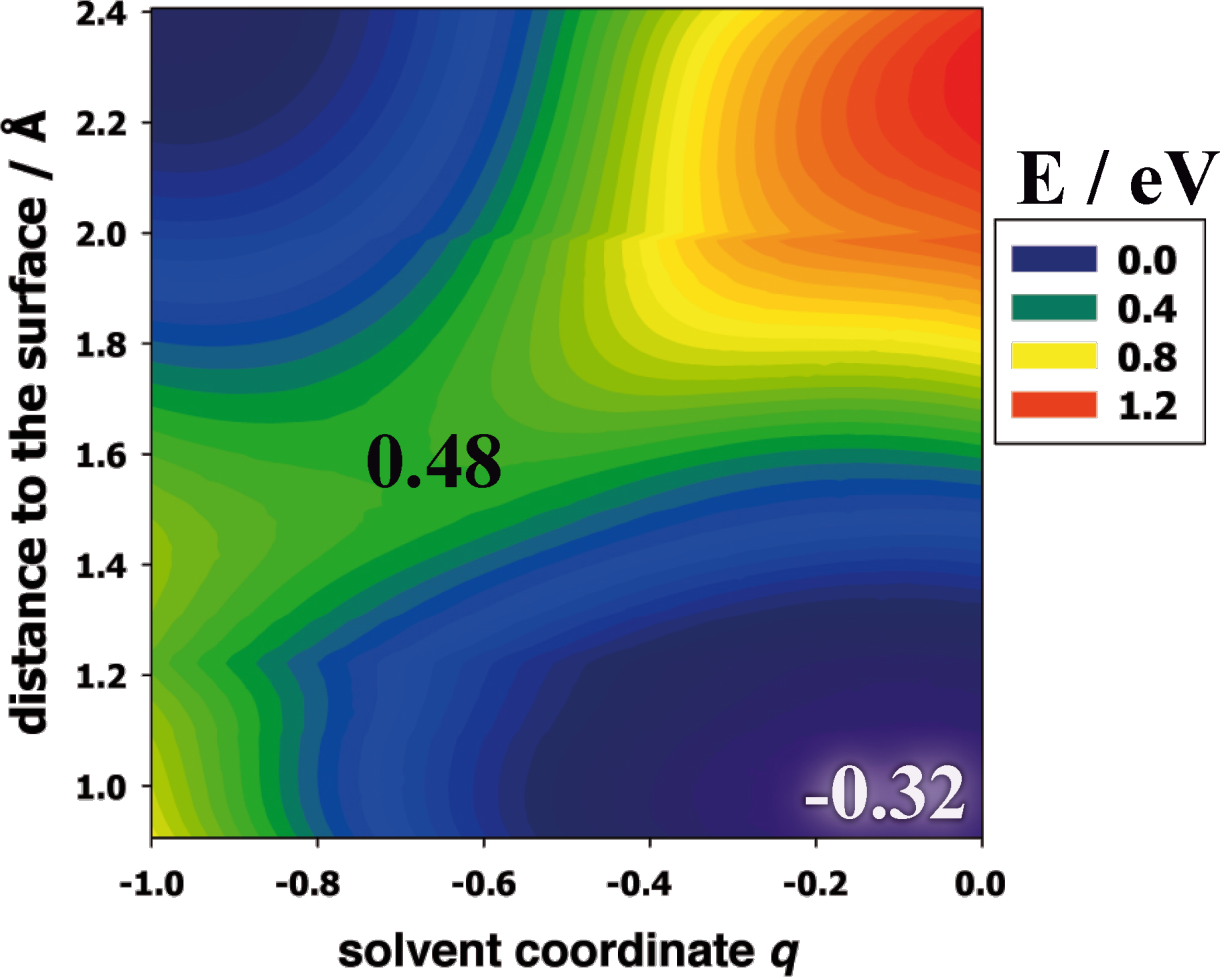
Free energy surface for the Volmer reaction on Ni(111) in acid solution at the equilibrium potential for hydrogen evolution.

We have calculated the free energy surface for hydrogen adsorption (Volmer reaction) on Ni(111) from our own theory. The calculations follow exactly our previous works [10,25], to which we refer for the details. In Figure 5 the surface has been plotted as a function of two coordinates: of the distance of the reactant from the surface, and of the solvent coordinate *q*, which characterizes the state of the solvent. In our normalization a solvent coordinate of value *q* indicates, that the solvent would be in equilibrium with a reactant of charge −*q*. Thus, the initial state of the reaction is a proton of charge one, which corresponds to *q* = −1, and is situated at large distances. The final state is an adsorbed, uncharged hydrogen atom with *q* = 0 on the metal surface. During the course of the reaction, the solvent is reorganized [6], and the system passes through a saddle point. At the equilibrium potential for hydrogen evolution the corresponding energy of activation is about 0.48 eV, which makes for a fast reaction. However, at this potential the reaction is exergonic by about 0.32 eV. Experimentally, on nickel and cobalt the Volmer–Heyrovsky mechanism has been found to operate, with the Heyrovsky step being rate-determining [28–29]. This agrees with our observations, that the Volmer step should be fast, and is perfectly in line with Sabatier’s principle.

## Final remarks

There is no doubt that Sabatier’s principle is sound, but the position of the d band and the details of its interaction with hydrogen are just as important. On the ascending branch of the plot, where Δ*G*_ad_ becomes more negative, these factors usually work in parallel, with the exception of the coinage metals, which have roughly the same rates because of the compensating effects discussed above. However, given the uncertainty of the experimental values, one can always draw a straight line starting somewhere near mercury and ending near platinum, which looks quite convincing even though the maximum is not at iridium, where it ought to be. The difficulty lies with the descending branch: When the oxide-covered metals are left out, only nickel and cobalt show the predicted decrease. On the other metals the reaction simply passes through intermediate states with higher energies.

A referee raised the valid question, why hydrogen evolution on oxide-covered metals has not been investigated systematically. There are several good reasons: (1) In an electrochemical environment, the oxide films on metal surfaces are not crystalline, but amorphous. In addition, they often incorporate OH and water. A good overview is given in [30]. (2) DFT studies on perfect oxides have shown, that often hydrogen is adsorbed as a proton, and is often incorporated into the film. WO_3_ is a good example for this effect [31]. (3) The experimental data are affected by the charge transport through the film, which is very difficult to correct for [32]. Therefore, for this class of electrodes it is impossible to relate DFT data for hydrogen adsorption with experimental data.

Besides the original work of Trasatti, several other volcano-type plots have appeared in the literature. There is no point in giving a complete list, so we mention a few that we think particularly valuable. The Nørskov group [7] was the first to use adsorption free energies calculated by DFT, and thus produced a more reliable and larger set of energies. The main difference between their plot and ours is, that we have added a few sp metals, used more recent data for Pt, Pd, and Ir, and left out the oxide-covered metals. Another volcano plot was presented by the same group in [33]. It was obtained by kinetic modelling based on the assumption that in all cases the Tafel reaction (Equation 2) is the rate-determining step, and that the Volmer reaction is always in equilibrium – as we have mentioned above, only Pt(111) and rhenium in acid solutions actually follow this path. Sheng et al. [34] have proposed a volcano plot for alkaline solutions. All of these plots lose their volcano shapes, once the oxide-covered metals are deleted.

Besides hydrogen adsorption energies, correlations have been proposed with a host of other metal characteristics: work function, latent heat of melting, lattice constants, etc. A fairly complete list has been given by Petrii and Tsirlina [11] and makes for an amusing read. They are not based on any sound principle like Sabatier’s, and it is not surprising that none of them has been successful [12].

For practical applications in fuel cells, the problem is not hydrogen oxidation but oxygen reduction, which is slow and inefficient. The full reduction involves four electron transfer steps, and possibly other chemical steps. The overall rate on a given substrate depends strongly on the pH value, and is also affected by anions. It is not surprising, that the details of the mechanism are still very much a subject of debate. Nevertheless, several attempts have been made to construct volcano plots for oxygen reduction as well. Really this topic is outside of the scope of this paper, so we just make a few brief comments meant as food for thought.

A principal difficulty is the lack of reliable data. Older data have been collected by Kinoshita [35], but the values obtained by different groups on similar systems differ widely. Therefore, it is not surprising that the volcano plot quoted most often is purely theoretical, calculated by the Nørskov group on the basis of a thermodynamic model for acid solutions [36]. Since this reaction contains so many steps, it is not clear which adsorption energy should be plotted on the x axis. This group has opted for the energy of adsorption of atomic oxygen; other candidates such as OH or OOH adsorption energies show decent linear correlations with oxygen adsorption.

In acid solutions, the first and rate-determining step is:

[6]
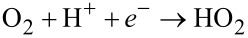


**Figure 6:**
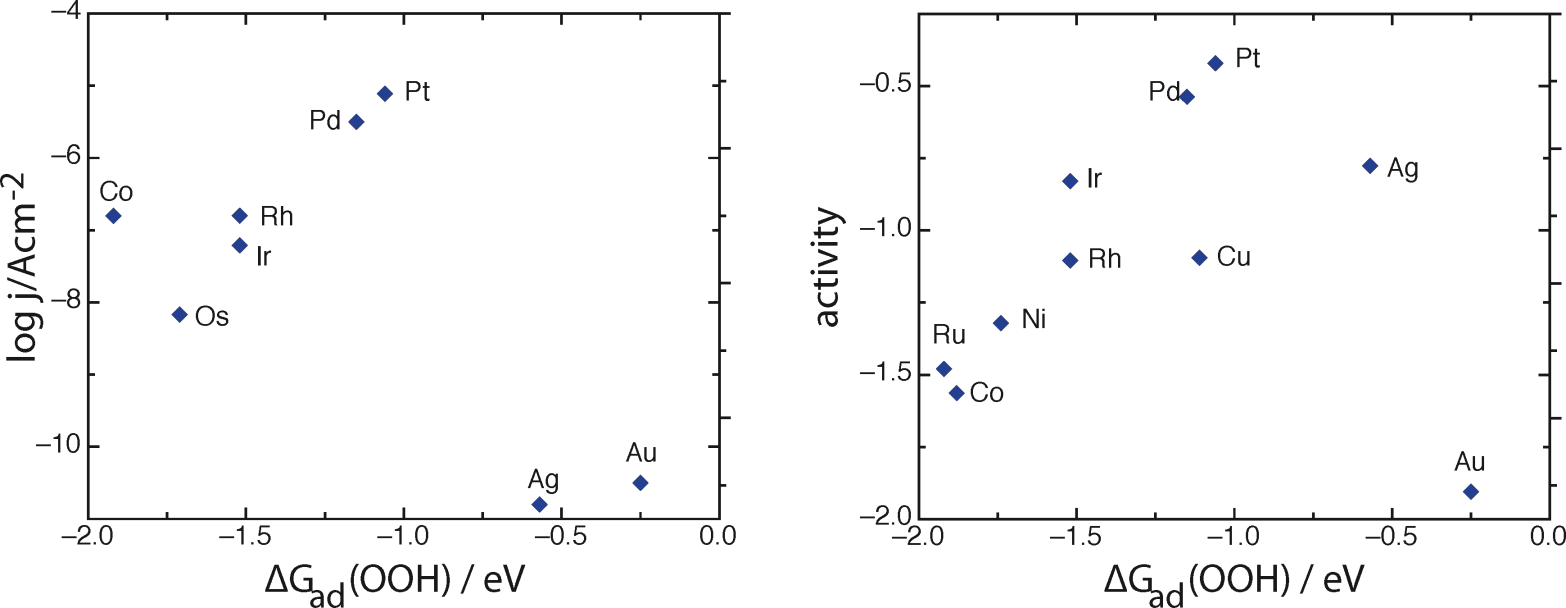
Oxygen reduction on various substrates in acid solutions. Left: logarithm of the current at 800 mV NHE in 85% phosphoric acid at 25°C plotted versus the adsorption energy of OOH on (111) surfaces; experimental data from Appleby [39], adsorption energies from [37]. Right: electrode activity (theoretical) for oxygen evolution in acid solutions versus OOH adsorption energy. The activity is proportional to the logarithm of the rate constant; the corresponding values have been taken from [36].

In the outer sphere mode this reaction has a standard equilibrium potential of −0.046 V SHE, which has to be compared with the standard potential for oxygen reduction at pH 0, 1.229 V SHE. Obviously, on a good catalyst for this reaction the adsorption energy must be of the order of 1 eV – which is exactly the energy of adsorption of OOH on Pt(111) [37]. We have replotted the theoretical activities as calculated by Nørskov et al. [36] in Figure 6 as a function of the OOH adsorption energy, which seems the more natural descriptor to us. The resulting plot still looks more or less like a volcano, but it is not as nice as in the original paper, since the correlation between O and OOH adsorption energies is not perfect. As for experimental data, there is one consistent set of data for the oxygen reduction in 85% phosphoric acid; this was once a popular solution because of the phosphoric acid fuel cell. We have plotted the corresponding data in the same figure. There are some obvious similarities and differences, which we shall not discuss. For obvious reasons the theoretical points form the nicer volcano. Finally we remark, that a recent volcano plot correlating experimental data with OH adsorption energies is not convincing [38], because the experimental data mix results obtained in acid and in alkaline solutions, even though, for example, oxygen reduction on gold and silver are many orders of magnitude faster in alkaline than in acid solutions.

In summary: Volcano plots are a valiant attempt to understand catalytic reactions with the aid of a single descriptor, typically the energy of adsorption of a single intermediate. However, the kinetics of complex reactions are not so simple.

## Appendix

### Sources of the experimental data for hydrogen evolution

#### sp Metals

All values for alkaline solutions are from Petrii and Tsirlina [11]; values for acid solutions are from the same source and from Trasatti [5]. The latter values are systematically higher than the former for reasons explained above.

#### Coinage metals

Values for acid solutions are from Norskøv et al. [7]; for alkaline solutions they are from Sheng et al. [34].

#### d Metals

Values for alkaline solutions are again form Sheng et al. [34]. Those for acid solutions are from Norskøv et al. [7]; for Pt, Ir, Pd we have also taken values from Chen and A. Kucernak [21] and Gasteiger et al. [22]; the latter are higher than the older values for reasons explained above.
